# Supplementary material for: In situ assembly of an injectable cardiac stimulator
Source: Nat Commun. 2024 Aug 8;15:6774. doi: 10.1038/s41467-024-51111-4 (PMC11310494; doi:10.1038/s41467-024-51111-4)
Supplement: Supplementary file 1 — Supplementary Information [file 41467_2024_51111_MOESM1_ESM.docx]

***Supplementary Information***

**In Situ Assembly of an Injectable Cardiac Stimulator**

Umut Aydemir^1^, Abdelrazek H. Mousa^2^, Cedric Dicko^3^, Xenofon Strakosas^4^, Muhammad Anwar Shameem^2^, Karin Hellman^1^, Amit Singh Yadav^1^, Peter Ekström^1^, Damien Hughes^1^, Fredrik Ek^1^, Magnus Berggren^4^, Anders Arner^5^, Martin Hjort^1^ and Roger Olsson^1,2^*

^1^Chemical Biology & Therapeutics, Department of Experimental Medical Science, Lund University, SE-221 84 Lund, Sweden,

^2^Department of Chemistry and Molecular Biology, University of Gothenburg, SE-405 30 Gothenburg, Sweden.

^3^Pure and Applied Biochemistry, Department of Chemistry, Lund University, SE-221 84 Lund, Sweden.

^4^Laboratory of Organic Electronics, Department of Science and Technology, Linköping University, SE-601 74 Norrköping, Sweden.

^5^Department of Clinical Sciences, Lund University, SE-221 84 Lund, Sweden.

*Corresponding author: Roger Olsson, roger.olsson@med.lu.se

**List of contents**

- *Immunohistofluorescence studies with zebrafish larvae*
- *Biomechanical analysis*
- *Adhesion analysis*
- *Supplementary references*

*Immunohistofluorescence studies with zebrafish larvae*

As positive controls for our immunohistochemistry studies, we employed anti-myeloperoxidase antibody staining (Abcam, # ab210563 , Rabbit polyclonal to myeloperoxidase, LOT# GR3443812-4) in zebrafish larvae. 5 dpf zebrafish larvae were anesthetized in 1.5 mM tricane solution for 10 minutes. Subsequently, the larvae were fixed by immersing them in 4 % PFA (Histolab, Phosphate buffered, HL 96753.1000) solution in 0.1 M phosphate buffer overnight. After fixation, the larvae were rinsed 3 x 1 h in PBS. The larvae were then permeabilized in PBS-TX for 3 hours at room temperature on a shake table. In this case, following permeabilization, the blocking step were followed by incubating larvae in 10 % goat normal serum in PBS-TX for 3 hours at room temperature on a shake table. Subsequently, the larvae were incubated in mouse anti-myeloperoxidase diluted 1:100 or 1:200 in PBS-TX-BSA. The larvae were incubated in 7 mL clear vials (Supelco 27150-U) with flat bottoms with intermittent agitation on a shake table. After the primary antibody incubation, the larvae were thoroughly rinsed in PBS-TX, 4 x 45 minutes at room temperature on a shake table. Then, the larvae were incubated in the secondary antibody, goat anti-rabbit IgG Alexa Fluor 546 conjugate (Invitrogen, LOT # 2539808) diluted 1:500 in PBS-Triton X-BSA, overnight at room temperature on a shake table while protecting the vials from light. Following the secondary antibody incubation, the larvae were rinsed 3 x 1 h in PBS-TX at room temperature on a shake table, followed by 3 x 30 minutes rinses in PBS on a shake table. Finally, the larvae were carefully transferred onto microscope slides, excess PBS was blotted and were covered with mounting medium ProLong^TM^ Gold antifade reagent with DAPI (Invitrogen, P36931). A cover glass was applied to seal the sample. 15 larvae were used (n=15) for whole mount immunofluorescence.



*Biomechanical Analysis*

**Analysis**: The data was analyzed according to methods described in supplementary reference 1 and 2.

**Analysis of the contact step**

The contact step is the first indentation of the pristine sample surface before doing any stress-relaxation study. The step informs on the direct elastic properties in indentation of the materials. The advantage of using the contact profile is that the samples are undisturbed and display a typical soft tissue elastic deformation. Shear-Modulus of heart found 2.0 +/- 0.7 while eBICS implanted heart found as 8.0 +/- 1.1. Young’s Modulus of heart found 8.0 +/- 1.1 while eBICS implanted heart found as 21.5 +/- 3.0. The moduli are significantly different (N=8, p-value=0.005, F-value = 23.4). Given the linear dependence between shear and Young’s moduli, the statistical test is valid for both.

**Analysis of the stress-relaxation profiles**

In this section, the samples were indented at specific depths (100 µm, 200 µm, and 300 µm) and left to relax. The combined indentation and relaxation are called ramp (ramp 1, 2, and 3). For each ramp, the following parameters were extracted:

Instantaneous shear modulus (kPa): The instantaneous modulus of eBICS refers to its stiffness or elasticity at a specific moment in time under dynamic or time-dependent loading conditions. ANOVA analysis of the heart and eBISC implanted heart showed no significant difference (regardless of the ramp) see Fig. Stable1.

Equilibriums shear modulus (kPa): The equilibrium modulus of eBICS refers to its stiffness or elasticity under static or constant loading conditions, where the applied stress or strain is constant and unchanged over an appropriate time. ANOVA analysis of the heart and eBICS implanted heart showed no significant difference (regardless of the ramp) see Fig. Stable 2.

*Effective relaxation time*: The time it takes for the force to reach 1/e of the start value. Effective relaxation time is an aggregated parameter that helps determine whether the relaxation process (viscoelastic or poroelastic) is fast or slow. ANOVA analysis of the heart and eBICS implanted heart showed no significant difference (regardless of the ramp) see Fig. Stable 3.

*Relaxation times(s)*: We estimated the storage modulus *G’* and the loss modulus *G’’* as a frequency function from the relaxation times. Using the simple equivalence, *G’* and *G’’* calculated from the relaxation times and shear moduli

$$G^{'}\left( \omega\right)=G_{\infty}+\sum_{1}^{N} G_{i}\frac{\left( \omega\tau_{i} \right)^{2}}{1+ \left( \omega\tau_{i} \right)^{2}}$$

$$G^{''}\left( \omega\right)=G_{\infty}+\sum_{1}^{N} G_{i}\frac{\left( \omega\tau_{i} \right)}{1+ \left( \omega\tau_{i} \right)^{2}}$$

The dynamic response appears different but the statistical test implies that is non-significant, in difference to the static test. This is not surprising since the dynamic test corrects for any variability in the indentation rate. From the ANOVA analysis of the dynamic response as a function of 1.5 Hz (see Fig. Stable 4) the heart vs eBICS implanted heart found statistically insignificant (p-value=0.642). Modulus Types are different, as we observed from the plots. The storage modulus is higher than the loss modulus (by a factor of 2-3).

**Adhesion analysis**

Adhesive properties can be extracted using the indentation setup used to deduce material stiffness. Upon indentation, soft materials can display a pull-on adhesion and when the load is released a pull-off adhesion. Soft materials are suitable for these measurements since they create large contact areas at contact due to their low stiffness. Adhesive forces on stiff materials in dry conditions are generally too small to be detected by current nanoindentation testers. In Figure S9 the indentation setup and the two adhesions for the eBICS on the zebrafish heart is presented.

Using the Johnson, Kendall and Roberts theory (JKR)^3^ to describe the contact of soft materials, one can derive the following equation for the pull-off force:

$$F_{JKR}=\frac{3}{2}\pi RW_{12}=F_{ad}$$

Where the force of adhesion (*F_ad_*) is proportional to the indenter radius (*R*) and the surface energy (*W_12_*). Here *R*=0.5mm. In Table S5 we estimate the force of adhesion from the contact mechanics and calculated the surface energy.

We kept the experimental conditions the same for the native heart and eBICS on heart (e.g. same indenter, same temperature, etc) allowing for direct comparisons. We observe that the force of adhesion is similar for the heart alone and the eBICS on the heart.

Interestingly, the adhesion is observed mainly in dry conditions. It is much less pronounced – if present at all – when the sample is fully immersed in liquid. This indicates that the adhesive forces are related to capillary forces due to water vapor condensation at the indenter-sample interface. These forces are not present once the indenter-sample interface is in liquid since no condensation occurs.

**
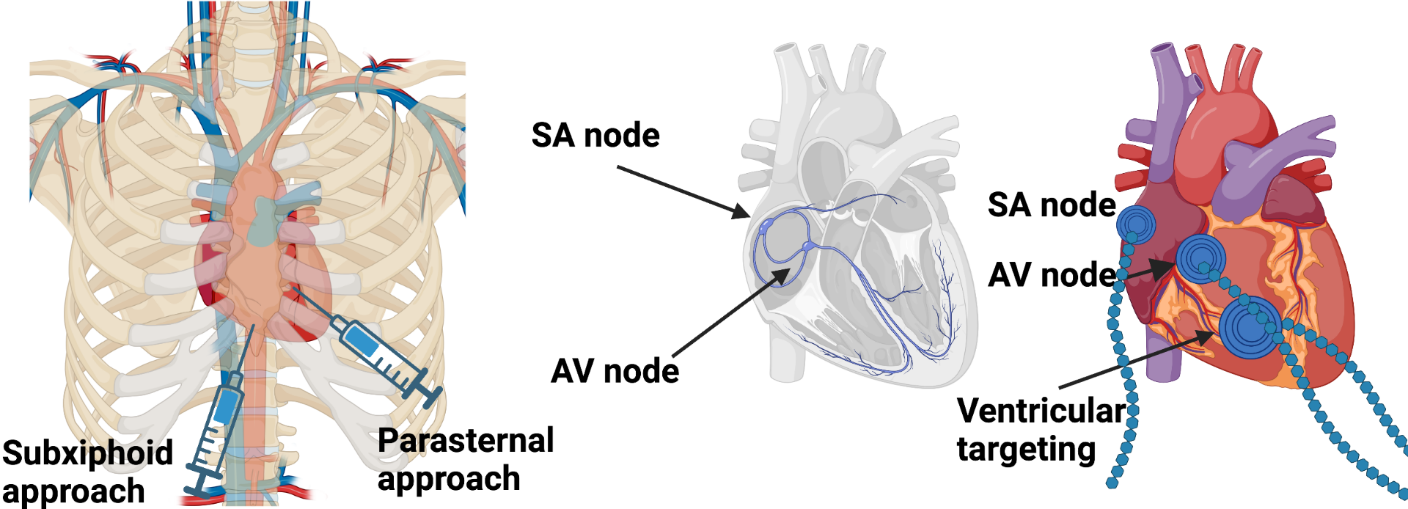
**

Fig. S1. Proposed guidance of BICS into position using anatomical landmarks. Figure created with BioRender.com released under a Creative Commons Attribution-NonCommercial-NoDerivs 4.0 International license (https://creativecommons.org/licenses/by-nc-nd/4.0/deed.en)


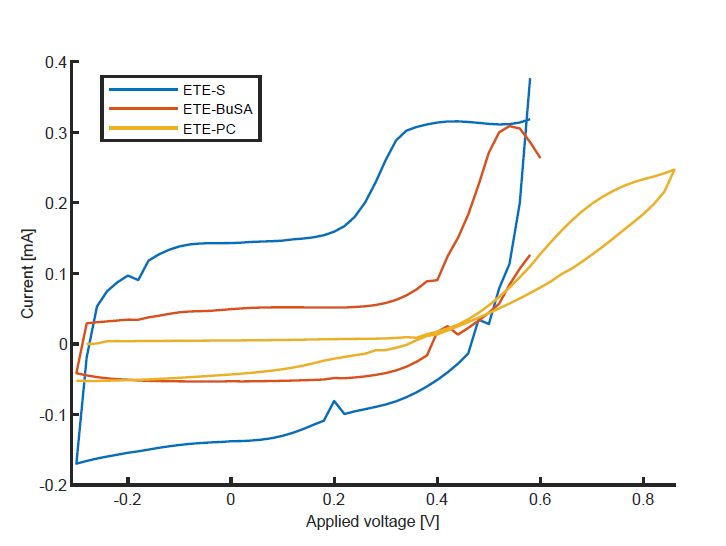


Fig. S2. Cyclic voltammetry measured for ETE-S, ETE-BuSA, and ETE-PC [7mg/ml] in NaCl [70mM]. Sweeps were obtained at 10 mV/s.

Fig. S3. Absorbance spectra of A5, ETE-BuSA, proBICS and eBICS.


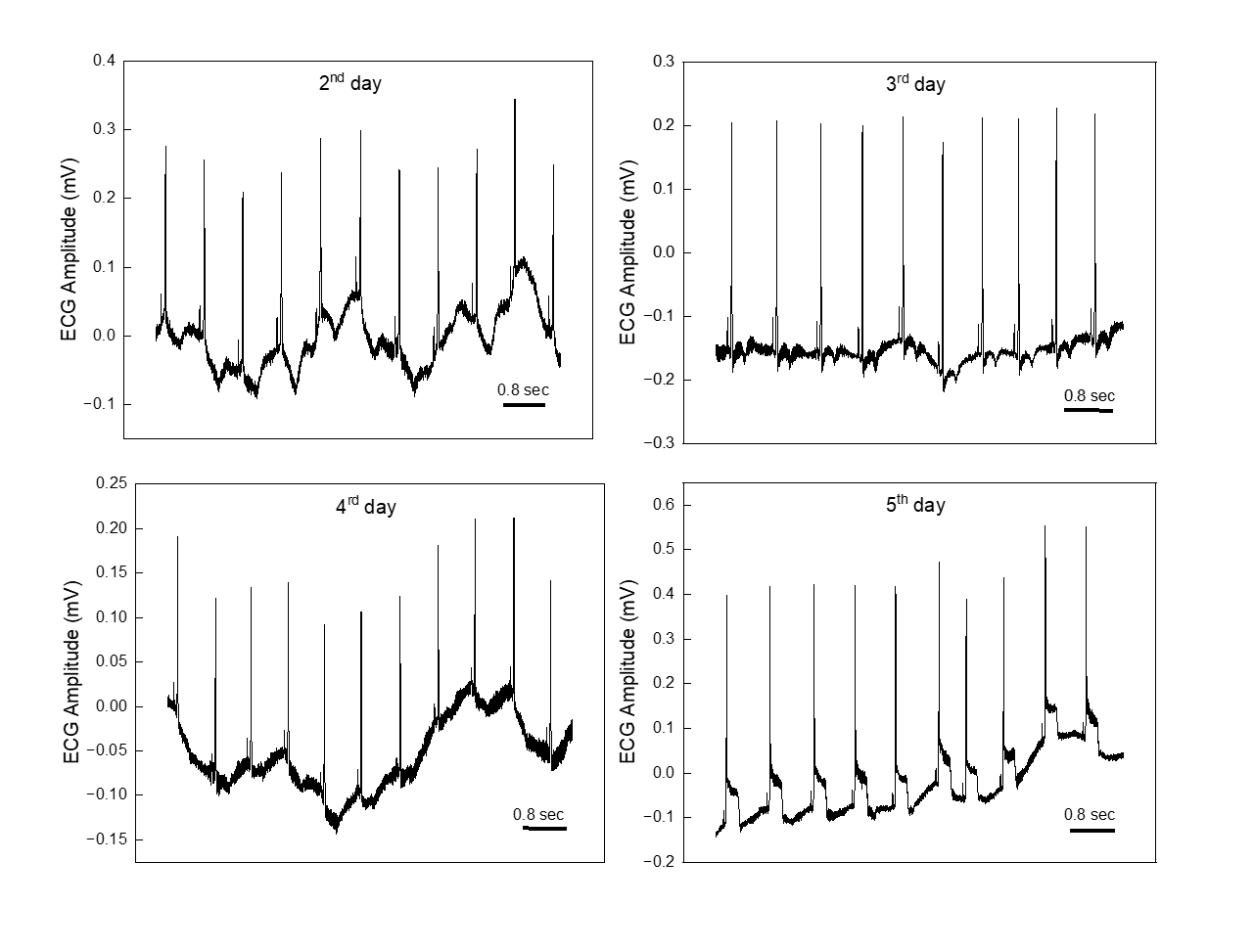


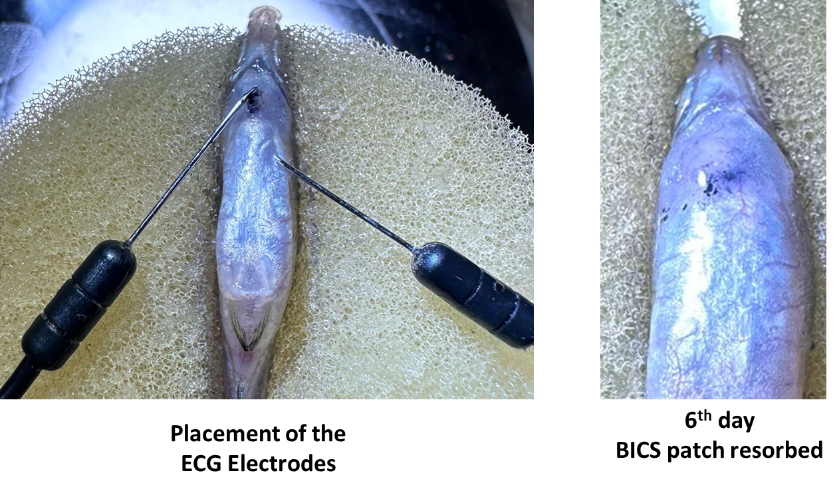


Fig. S4. ECG recordings from an individual zebrafish with eBICS installed, lasting 5 consecutive days.


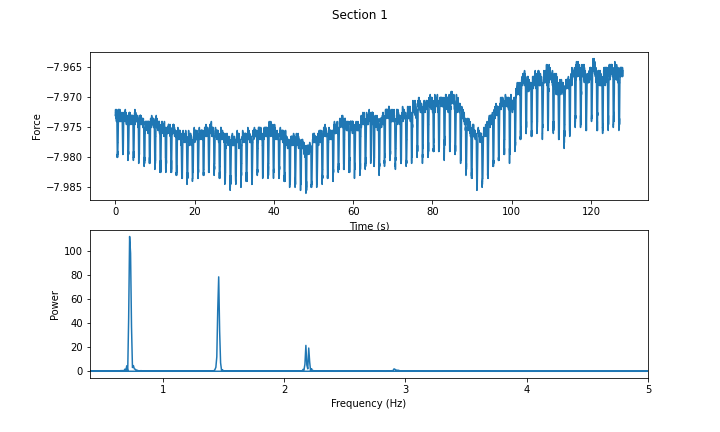


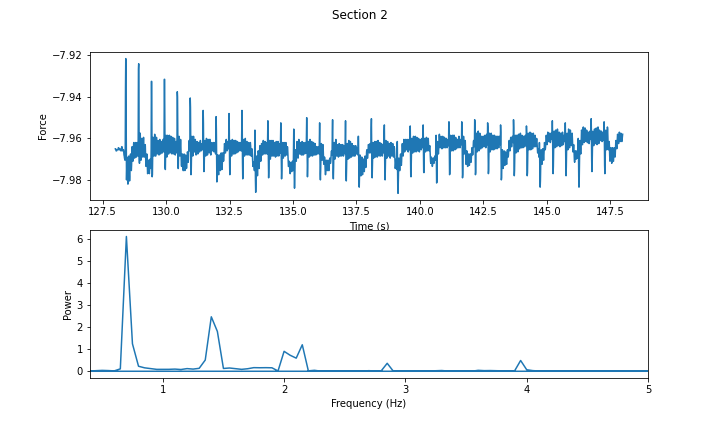


Fig. S5. Reference fish show no heart beat change. Control experiments conducted on fish without BICS showed no change in beating patterns when applying external stimulation pulses.


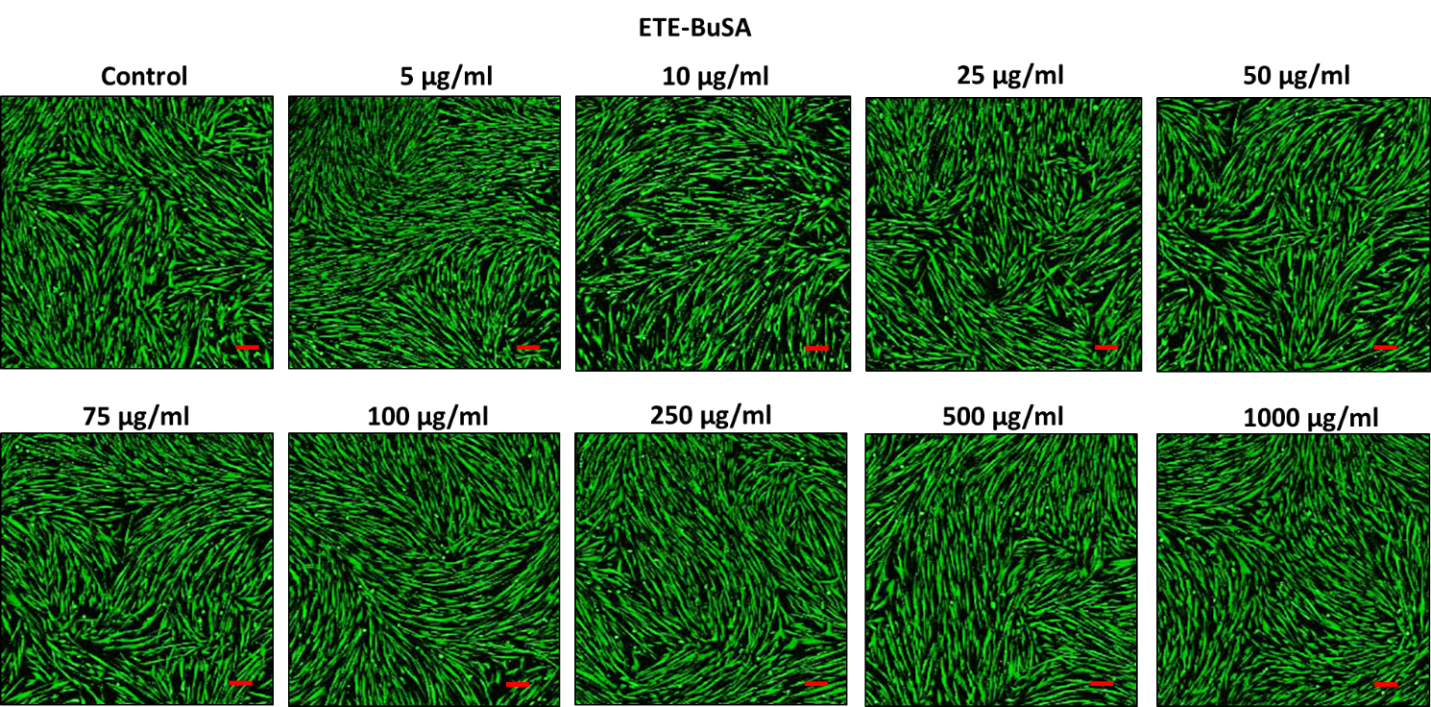


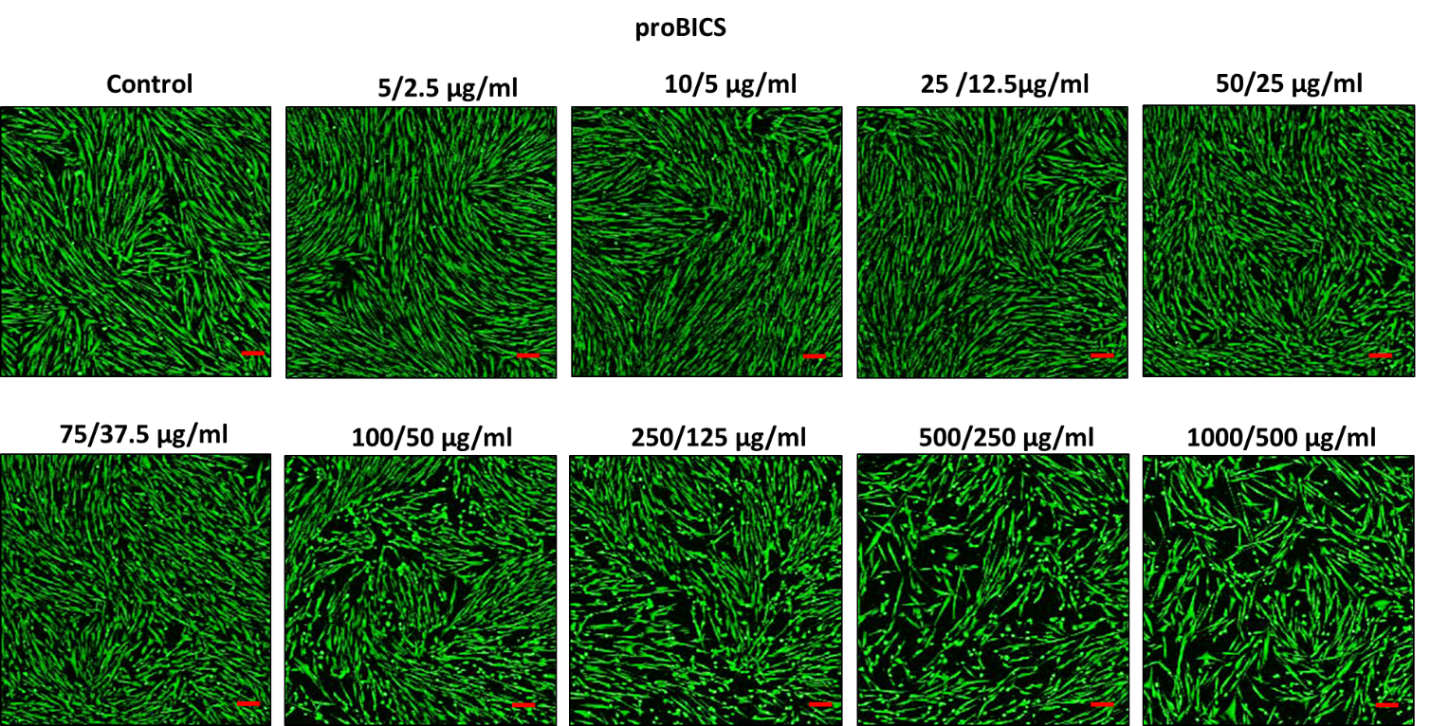


Fig. S6. Toxicity screen in an ex vivo cell culture. Calcein-AM staining of HFL-1 cells upon treatments of ETE-BuSA and proBICS. the density of stained cells was not altered by treatment with ETE-BuSA or proBICS at concentrations of up 1000 µg/mL for 24 hours of treatment.


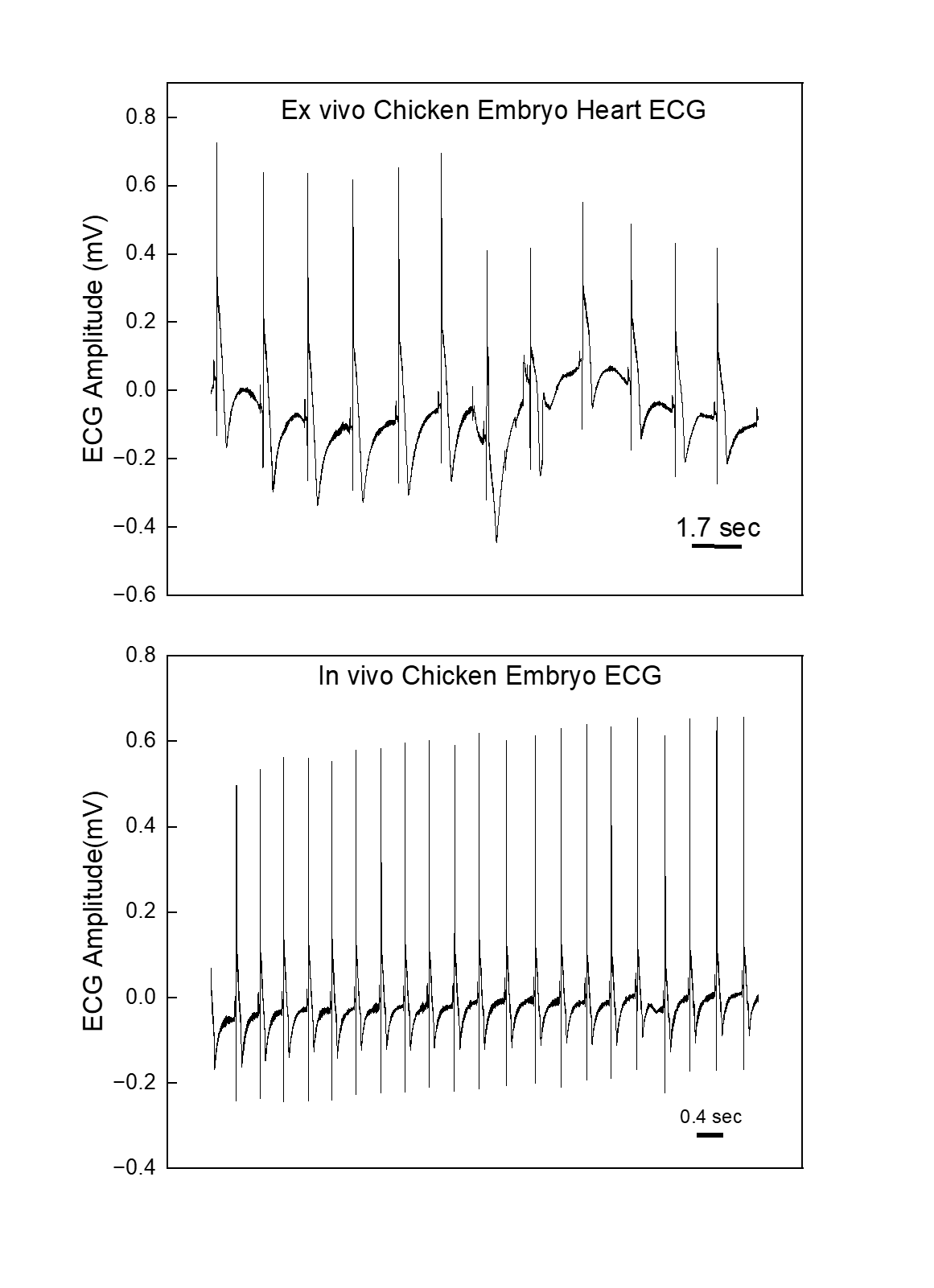


Fig. S7. Ex vivo and in vivo chicken embryo ECGs.

Fig. S8. In vivo BICS injected chicken embryo ECG.

**
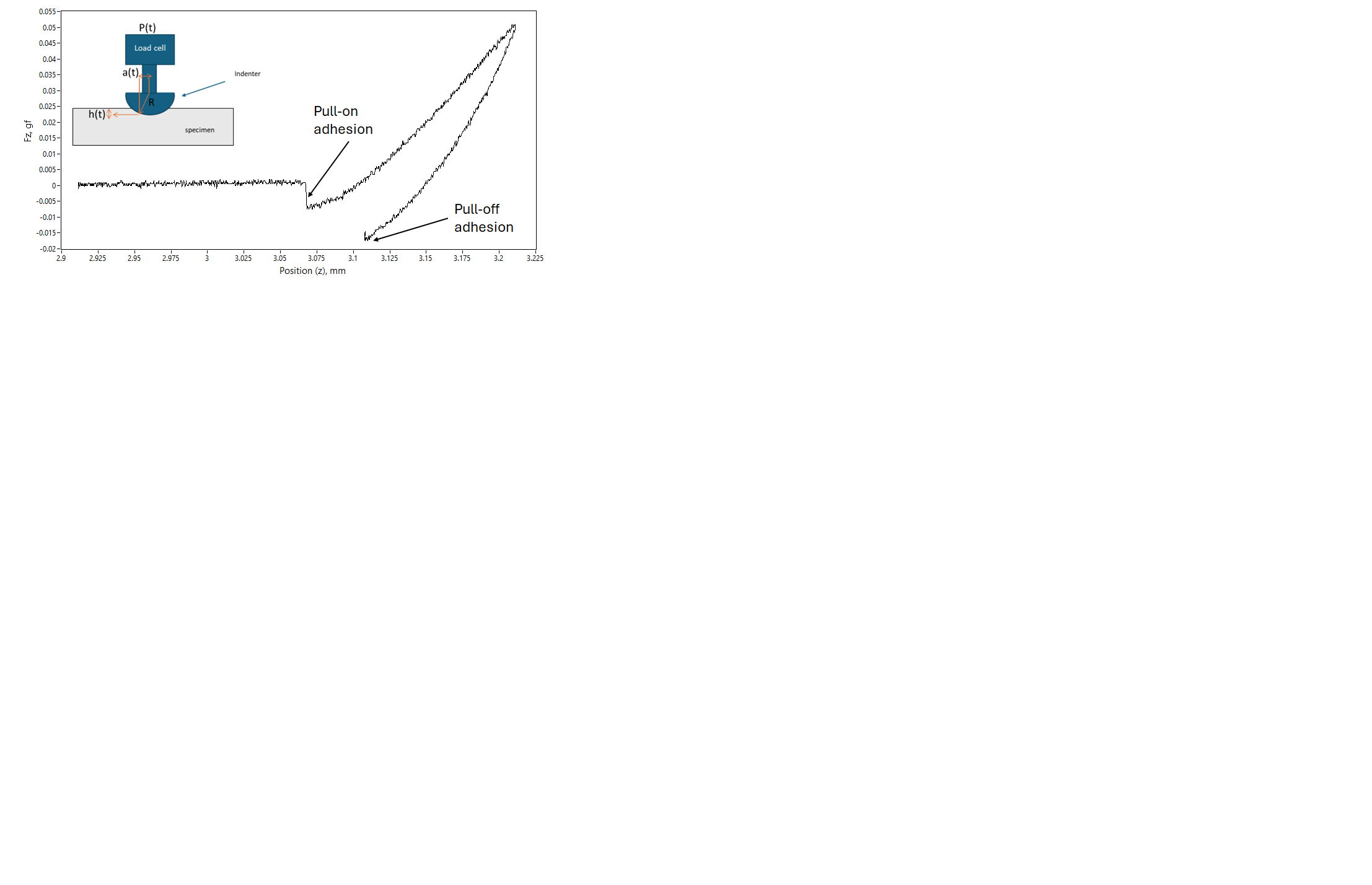
**

Fig. S9. The indentation setup and the two adhesions for the eBICS on the zebrafish heart. Contact mechanics for the conductive polymer is an initial pull-on adhesion followed by a pulled-off adhesion when the load is removed

**
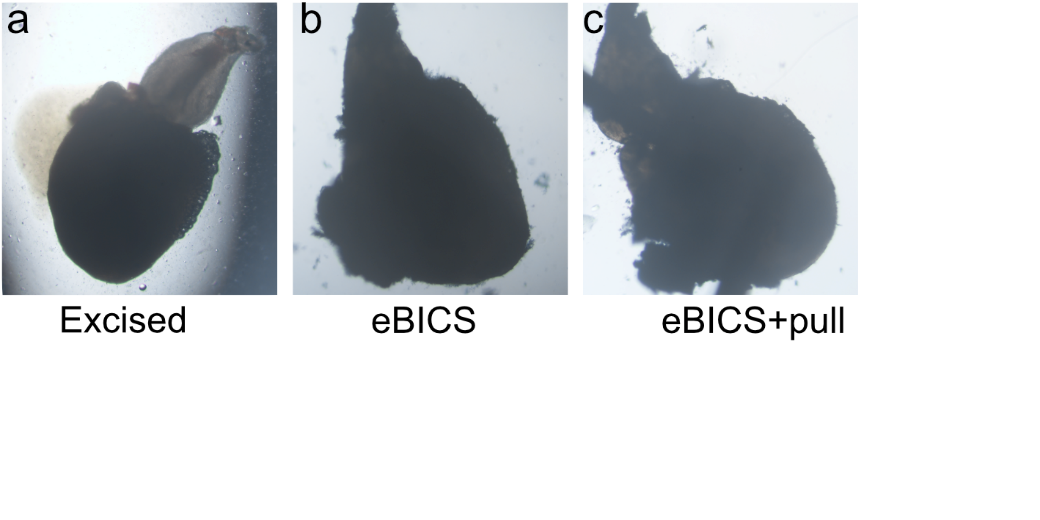
**

Fig. S10. eBICS adhesion on excised zebrafish heart after pulling. (a) Zebrafish heart post excision. (b) The heart after applying 5 µl proBICS in vitro and electrofunctionalization using 1 V. (c) The heart after extensive pulling using micromanipulators as shown in Supplemental movie 7. Please note that the eBICS still adheres to the heart.


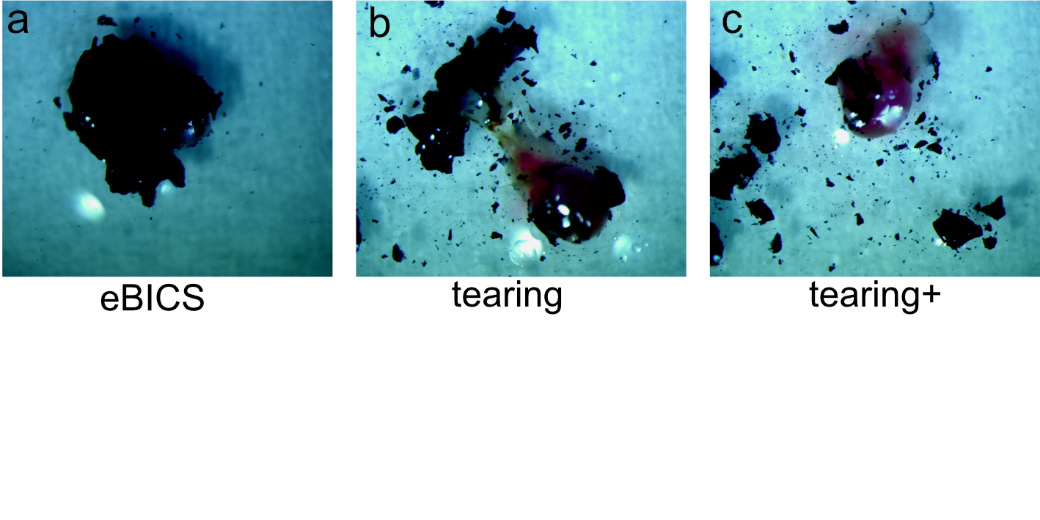


Fig. S11. eBICS adhesion on excised zebrafish heart after tearing. (a) Zebrafish heart post excision, completely covered by proBICS in vitro and electrofunctionalized using 1 V. (b) The heart after extensive tearing using tweezers. (c) The heart after tearing the heart apart using tweezers. Please note that the eBICS still adheres to the heart.


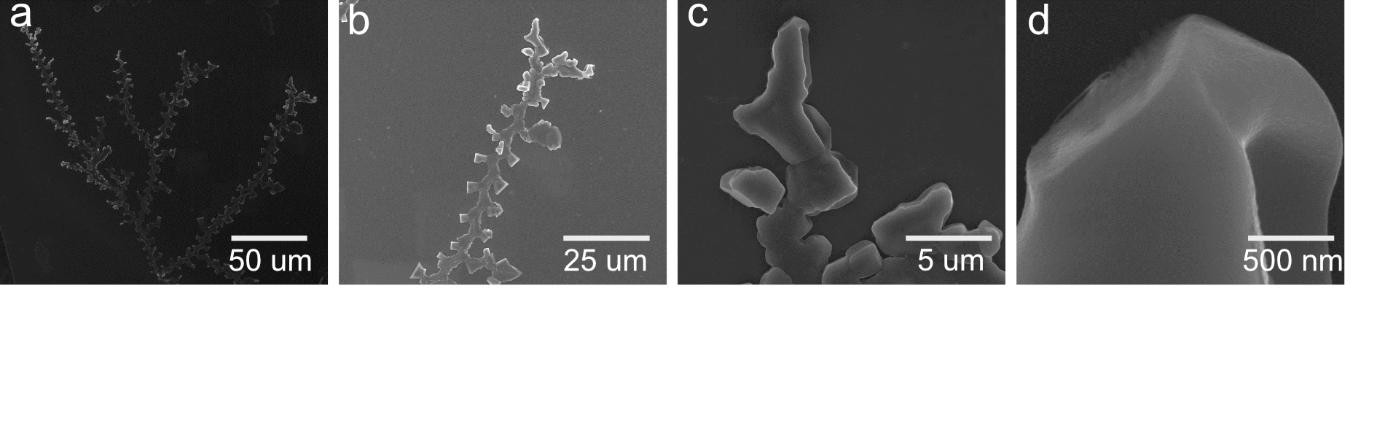


Fig. S12. Scanning electron micrographs depicting eBICS formed on a silicon wafer. (a–d) Micrographs from the same region at increasing magnifications. A droplet of proBICS was added onto a Si wafer and contacted by a Au coated W electrode (anode). An AgCl electrode was dipped into a PBS droplet which was in contact with the proBICS to allow electrofunctionalization at 1 V for 5min.After eBICS formation, excess salts from the PBS was rinsed away using 96% EtOH. Sample was then left to dry, coated with 4 nm Pt:Pd and imaged in SEM using 10 kV electron beam. Dendritic structures with some faceting were observed.


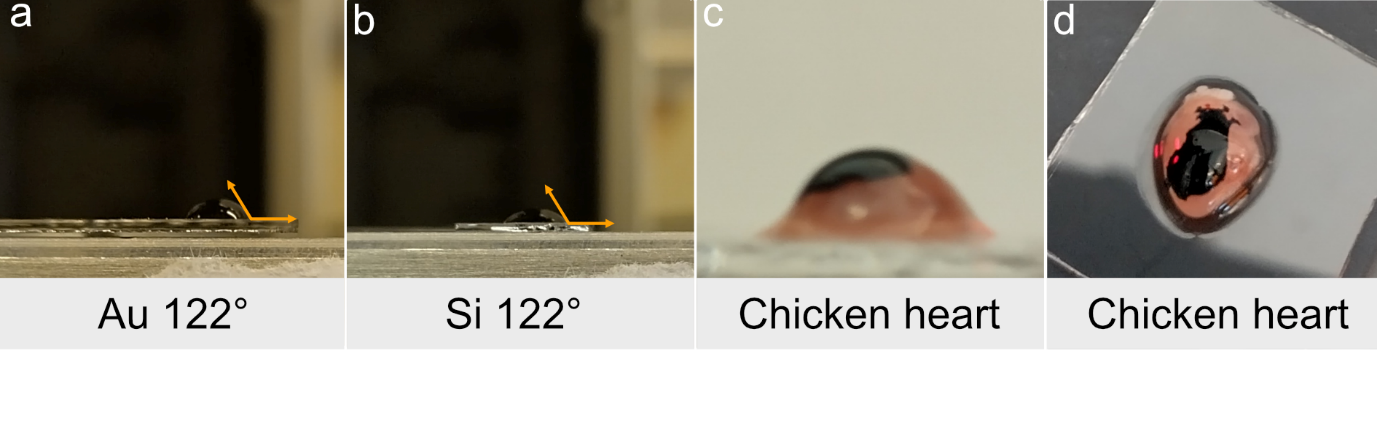


Fig. S13. Contact angle measurements. 10 µl proBICS was carefully pipetted onto a Au coated glass slide (a), a piece of Si wafer (b), and a beating, excised chicken heart EDD 14 (c, d). The proBICS wets the chicken heart without forming a well-defined droplet.

Fig. S14. Electrocardiogram obtained from an excised zebrafish heart with eBICS installed on it. After stimulation (middle segment), the heart goes back to beat at it’s native frequency, 0.8Hz.


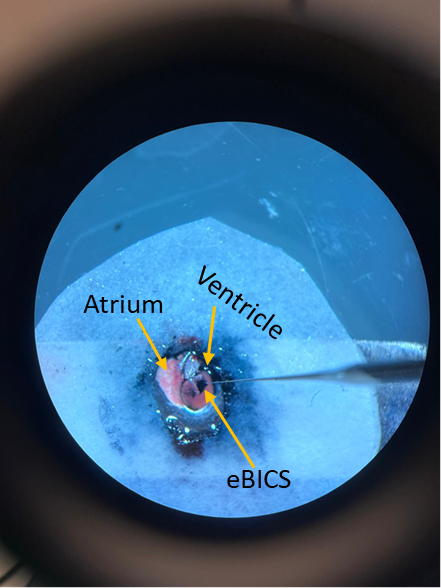


Fig. S15. Excised, beating zebrafish heart after eBICS installation. After electrofunctionalization, there were no signs of abnormalities or tissue damage to the atrium nor the ventricle. In the right part of the image, the injection capillary can be seen to the right.


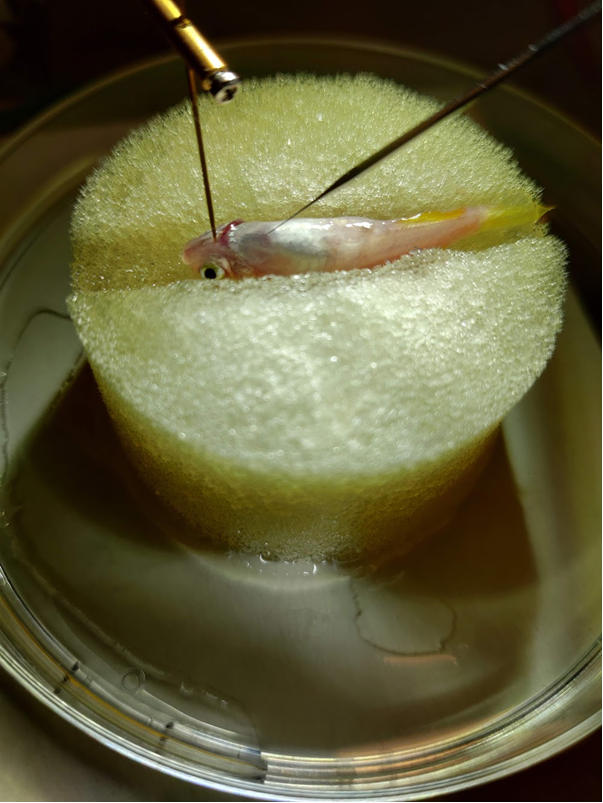


Fig. S16. Anesthetized zebrafish placed in a sponge during microinjection of the proBICS solution.

| ANOVA – Instantaneous Shear Modulus | | | | | | | | | | | |
| --- | --- | --- | --- | --- | --- | --- | --- | --- | --- | --- | --- |
|  | | Sum of Squares | | df | | Mean Square | | F | | p | |
| Heart vs Heart eBICS |  | 0.0322 |  | 1 |  | 0.0322 |  | 0.0272 |  | 0.873 |  |
| Ramp |  | 2.9304 |  | 2 |  | 1.4652 |  | 1.2385 |  | 0.335 |  |
| Heart vs Heart eBICS ✻ Ramp |  | 0.0246 |  | 2 |  | 0.0123 |  | 0.0104 |  | 0.990 |  |
| Residuals |  | 10.6473 |  | 9 |  | 1.1830 |  |  |  |  |  |
|  | | | | | | | | | | | |

Table S1. One-way ANOVA analysis of the instantaneous shear moduli of heart and eBICS implanted heart. The analysis showed no significant difference (regardless of the ramp) between the measurement groups.

| ANOVA – Equilibrium Shear Modulus | | | | | | | | | | | |
| --- | --- | --- | --- | --- | --- | --- | --- | --- | --- | --- | --- |
|  | | Sum of Squares | | df | | Mean Square | | F | | p | |
| Heart vs Heart eBICS |  | 0.0348 |  | 1 |  | 0.0348 |  | 0.0415 |  | 0.843 |  |
| Ramp |  | 1.8250 |  | 2 |  | 0.9125 |  | 1.0902 |  | 0.377 |  |
| Heart vs Heart eBICS ✻ Ramp |  | 0.4383 |  | 2 |  | 0.2191 |  | 0.2618 |  | 0.775 |  |
| Residuals |  | 7.5328 |  | 9 |  | 0.8370 |  |  |  |  |  |
|  | | | | | | | | | | | |

Table S2. One-way ANOVA analysis of the equilibrium shear moduli of heart and eBICS implanted heart. The analysis showed no significant difference (regardless of the ramp) between the measurement groups.

| ANOVA – Effective Relaxation | | | | | | | | | | | |
| --- | --- | --- | --- | --- | --- | --- | --- | --- | --- | --- | --- |
|  | | Sum of Squares | | df | | Mean Square | | F | | p | |
| Heart vs Heart eBICS |  | 0.702 |  | 1 |  | 0.702 |  | 2.35e-4 |  | 0.988 |  |
| Ramp |  | 1853.080 |  | 2 |  | 926.540 |  | 0.310 |  | 0.741 |  |
| Heart vs Heart eBICS ✻ Ramp |  | 3634.899 |  | 2 |  | 1817.449 |  | 0.608 |  | 0.565 |  |
| Residuals |  | 26902.046 |  | 9 |  | 2989.116 |  |  |  |  |  |
|  | | | | | | | | | | | |

Table S3. One-way ANOVA analysis of the effective relaxation of heart and eBICS implanted heart. The analysis showed no significant difference (regardless of the ramp) between the measurement groups.

| ANOVA – Frequency 1.5 Hz | | | | | | | | | | | |
| --- | --- | --- | --- | --- | --- | --- | --- | --- | --- | --- | --- |
|  | | Sum of Squares | | df | | Mean Square | | F | | p | |
| Heart vs Heart eBICS |  | 0.453 |  | 1 |  | 0.453 |  | 0.240 |  | 0.642 |  |
| ModulusType |  | 11.736 |  | 1 |  | 11.736 |  | 6.214 |  | 0.047 |  |
| SampleType ✻ ModulusType |  | 0.438 |  | 1 |  | 0.438 |  | 0.232 |  | 0.647 |  |
| Residuals |  | 11.333 |  | 6 |  | 1.889 |  |  |  |  |  |
|  | | | | | | | | | | | |

Table S4. One-way ANOVA analysis of the dynamic response to applied 1.5 Hz of heart and eBICS implanted heart. The analysis showed no significant difference (regardless of the ramp) between the measurement groups

|  | | *F_ad_* - Pull-off force (µN) | | Surface energy *W_12_* (mJ/m^2^) | |
| --- | --- | --- | --- | --- | --- |
| Heart |  | -143.3 ± 10.3 |  | 60.8 ± 4.4 |  |
| Heart eBICS |  | -145.4 ± 9.1 |  | 61.7 ± 3.9 |  |

Table S5. Adhesion properties for zebrafish heart and eBICS-heart as measured using a microindenter.

**Supplementary References**

1. Wang, M. *et al.* Characterizing poroelasticity of biological tissues by spherical indentation: An improved theory for large relaxation. *Journal of the Mechanics and Physics of Solids* **138**, 103920 (2020).

2. Babaei, B., Davarian, A., Pryse, K. M., Elson, E. L. & Genin, G. M. Efficient and optimized identification of generalized Maxwell viscoelastic relaxation spectra. *Journal of the Mechanical Behavior of Biomedical Materials* **55**, 32–41 (2016).

3. Johnson KL, Kendall K, Roberts AD., Surface energy and the contact of elastic solids. *Proc. R. Soc. Lond. A* 1971
