# Supplementary material for: In situ assembly of an injectable cardiac stimulator
Source: Nat Commun. 2024 Aug 8;15:6774. doi: 10.1038/s41467-024-51111-4 (PMC11310494; doi:10.1038/s41467-024-51111-4)
Supplement: Supplementary file 3 — Description Of Additional Supplementary File [file 41467_2024_51111_MOESM3_ESM.pdf]

Description of Additional supplementary file

Movie S1. Ex vivo electrical stimulation of zebrafish heart through strategically placed eBICS, touching the atrium. Increase in heart rhythm was observed with supplied electrical pulses. Electrical stimulation was performed using 4 V square voltage pulses with 10 milliseconds pulse width supplied by a Grass S48 Stimulator (AstroMed).

Movie S2. Fish swimming with a normal behavior after 1 hpi (hour post injection). The anesthetization, operation and stimulation procedures are well tolerated by the fish. No aberrant swimming pattern was observed to attribute a sign of stress.

Movie S3. Ex vivo electrical stimulation of chicken heart through strategically placed eBICS, touching the atrium. An increase in heart rhythm was observed with the supplied electrical pulses. Electrical stimulation was performed using 4 V square voltage pulses with 10 milliseconds pulse width supplied by a Grass S48 Stimulator (AstroMed).

Movie S4. Control experiment without eBICS, Ex vivo electrical stimulation of chicken heart through metal electrodes. When eBICS is not touching the heart, no activation with electrical pulses was observed.

Movie S5. Real time video depicting an eBICS coated zebrafish heart undergoing extensive hydrodynamic forces as it is being forced up and down the tip of a Pasteur pipette. Movie collected using a OnePlus mobile phone. Please note that the eBICS remains on the heart after the process.

Movie S6. Real time video depicting an eBICS coated zebrafish heart being torn to pieces by two tweezers. Movie collected using a OnePlus mobile phone. Please note that the eBICS remains on the heart after the process.

Movie S7. Real time video depicting an eBICS coated zebrafish heart being pulled and pressed using two micromanipulators. Movie obtained using a 4x objective in a Nikon Eclipse FN1 microscope. Please note that the eBICS remains on the heart after the process.
